# Porcine epidemic diarrhea virus inhibits dsRNA-induced interferon-β production in porcine intestinal epithelial cells by blockade of the RIG-I-mediated pathway

**DOI:** 10.1186/s12985-015-0345-x

**Published:** 2015-08-18

**Authors:** Liyan Cao, Xuying Ge, Yu Gao, Georg Herrler, Yudong Ren, Xiaofeng Ren, Guangxing Li

**Affiliations:** College of Veterinary Medicine, Northeast Agricultural University, 59 Mucai Street, Xiangfang District, Harbin, 150030 China; Institute of Virology University of Veterinary Medicine, BÜnteweg 17, D-30559 Hannover, Germany; College of Electrical and Information, Northeast Agricultural University, Harbin, 150030 China

## Abstract

**Background:**

The lack of optimal porcine cell lines has severely impeded the study and progress in elucidation of porcine epidemic diarrhea virus (PEDV) pathogenesis. Vero cell, an African green monkey kidney cell line, was often used to isolate and propagate PEDV. Nonetheless, the target cells of PEDV in *vivo* are intestinal epithelial cells, during infection, intestinal epithelia would be damaged and resulted in digestive disorders. The immune functions of porcine epithelial cells and interactions with other immune cell populations display a number of differences compared to other species. Type I interferon (IFN) plays an important role in antiviral immune response. Limited reports showed that PEDV could inhibit type I interferon production. In this study, porcine small intestinal epithelial cells (IECs), the target cells of PEDV, were used as the infection model *in vitro* to identify the possible molecular mechanisms of PEDV-inhibition IFN-β production.

**Results:**

PEDV not only failed to induce IFN-β expression, but also inhibited dsRNA-mediated IFN-β production in IECs. As the key IFN-β transcription factors, we found that dsRNA-induced activation of IFN regulatory factor 3 (IRF-3) was inhibited after PEDV infection, but not nuclear factor-kappaB (NF-κB). To identify the mechanism of PEDV intervention with dsRNA-mediated IFN-β expression more accurately, the role of individual molecules of RIG-I signaling pathway were investigated. In the upstream of IRF-3, TANK-binding kinase 1 (TBK1)-or inhibitor of κB kinase-ε (IKKε)-mediated IFN-β production was not blocked by PEDV, while RIG-I-and its adapter molecule IFN-β promoter stimulator 1 (IPS-1)-mediated IFN-β production were completely inhibited after PEDV infection.

**Conclusion:**

Taken together, our data demonstrated for the first time that PEDV infection of its target cell line, IECs, inhibited dsRNA-mediated IFN-β production by blocking the activation of IPS-1 in RIG-I-mediated pathway. Our studies offered new visions in understanding of the interaction between PEDV and host innate immune system.

## Background

Porcine epidemic diarrhea virus (PEDV) is an enveloped, single-stranded, RNA virus of *Coronaviridae* family, which is the main etiological agent of severe diarrhea in pigs of all ages and fatality in neonates [1]. Outbreaks of porcine epidemic diarrhea (PED) have received extensive attention for the considerable economic losses to the swine industry worldwide. Great advances have been made in elucidation of the molecular epidemiology, diagnosis, prevention, and treatment of PED [2]. Recently, coronavirus interaction with host innate immune system has been a hot research field. Previous studies indicated that transmissible gastroenteritis virus (TGEV) infection enhanced type I interferon expression and its protein 7 modulated type I IFN expression [3, 4]. For mouse hepatitis virus (MHV), IFN production among different cell populations varied due to their diverse susceptibility to this virus [5–9]. Furthermore, both severe acute respiratory syndrome coronavirus (SARS-CoV) and Middle East respiratory syndrome coronavirus (MERS-CoV) do not induce type I IFN (IFN-α/β) activation [10–12]. So far, limited reports showed that PEDV could inhibit type I interferon production [13, 14].

During viral infection and replication, the host innate immune response is the first line of defense; therefore, the ability of viruses to suppress or avoid this response is crucial for their pathogenic potential. IFN-α/β is an essential element of the host innate immune response against viral infections. Double-stranded RNA (dsRNA), the replicative intermediate of most viruses, is a potent inducer of IFN-β, which is recognized as a pathogen-associated molecular pattern (PAMP) by host pattern recognition receptors (PRRs). Two of major PRRs, retinoic acid-inducible gene I (RIG-I) and melanoma differentiation-associated gene 5 (MDA5) detect dsRNA in the cytoplasm [15]. Following dsRNA binding, RIG-I and MDA5 recruit corresponding adapter protein IFN-β promoter stimulator 1 (IPS-1) that, in turn, activate downstream signaling of TANK-binding kinase 1 (TBK1) and inhibitor of κB kinase-ε (IKKε) transduction, leading to the activation of transcription factor IFN regulatory factor 3 (IRF-3) and nuclear factor-kappaB (NF-κB). Activated IRF-3, and NF-κB bind to IFN-β enhancer and initiate IFN-β transcription [16].

Vero cell, an African green monkey kidney cell line, was often used to isolate and propagate PEDV [17]. However, it was often considered that Vero cells might lack genetic component necessary for IFN production [18–20]. Porcine intestinal epithelial cells (IECs) are thought to the target cells of PEDV, which play an important role in the activation of host immune responses by induction of key signaling molecules, including cytokines, surface molecules, and chemokines during microoganism invasion [21, 22]. In the present study, to determine if PEDV infection suppresses IFN-β activation, we chose IECs as an infection model to research the molecular mechanisms of PEDV infection and the host antiviral innate immune response. Our results clearly suggested that PEDV prevented dsRNA-induced IFN-β synthesis by blocking RIG-I-mediated pathways.

## Results and discussion

### PEDV failed to induce IFN-β expression and inhibited poly (I:C)-mediated IFN-β production in IECs

**Fig. 1:**
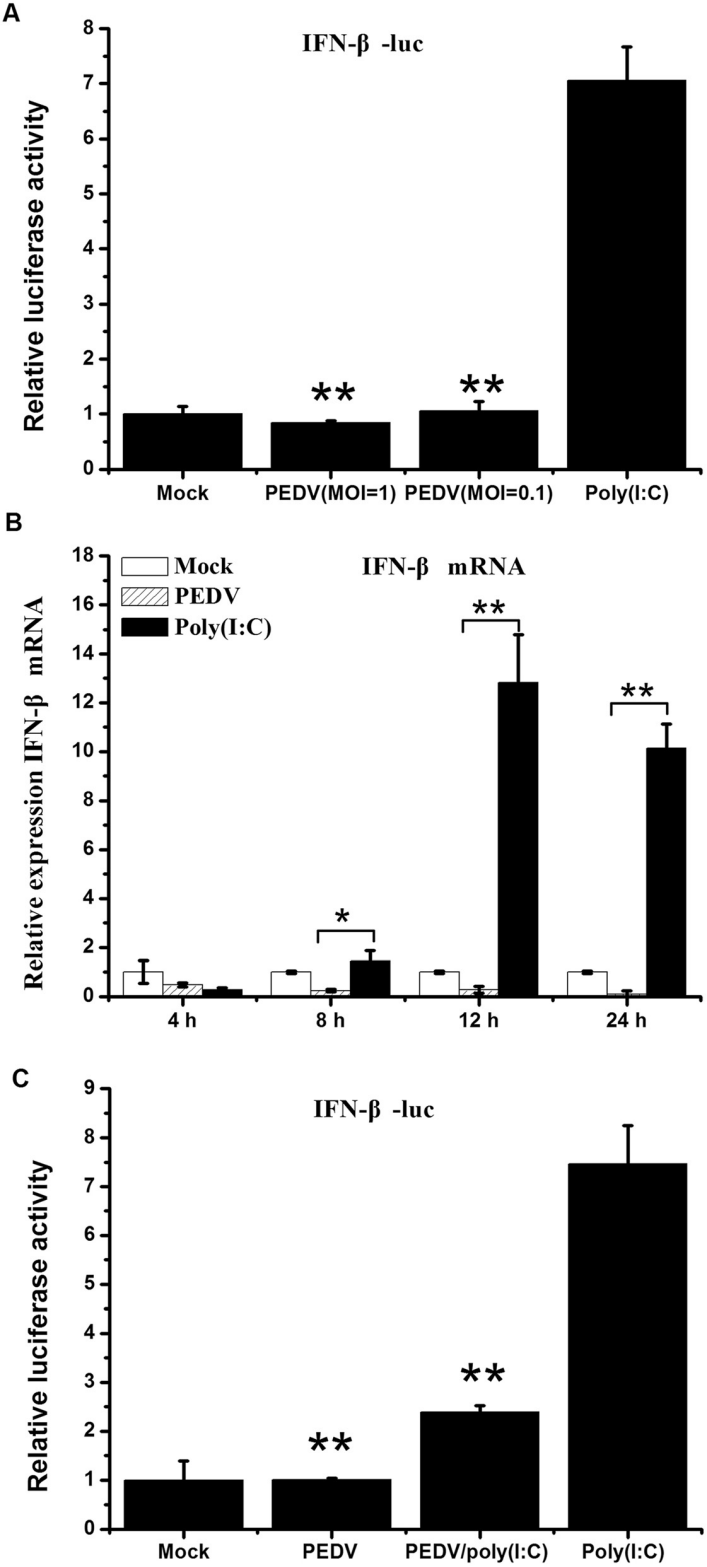
PEDV does not induce IFN-β production and inhibits poly (I:C)-mediated IFN-β induction. **a** IECs were cotransfected with IFN-β-Luc and phRL-TK, then infected with PEDV at an MOI of 1 and 0.1 for 24 h. Cells were retransfected with poly (I:C) as a positive control. After 12 h, the cells were harvested and subjected to a dual-luciferase assay. **b** IECs were infected with PEDV at an MOI of 1, mock-infected as a negative control, or transfected with poly (I:C) as a positive control. At the indicated time points, total RNA was extracted and IFN-β and β-actin mRNA were subjected to real-time PCR. RNA expression levels were normalized to β-actin. **c** In contrast to **a**, IECs were first mock-infected or infected with PEDV at an MOI of 1 for 12 h and then cotransfected with IFN-β-Luc and phRL-TK for 24 h. Cells were retransfected with or without poly (I:C) for an addition 12 h, harvested, and then subjected to a dual-luciferase assay. All data are expressed as means ± SD of 3 independent experiments. *p < 0.05; **p < 0.01 as compared with poly (I:C)

Type I IFNs (IFN-α/β) are critical to the host antiviral innate immune response. However, there is no evidence suggesting that IECs produce type I IFNs in response to PEDV infection. Previous studies have showed that PEDV could be propagated in IECs [ 23, 24,]. To confirm whether PEDV infection could induce IFN-β production in IECs or not, we transiently cotransfected the IFN-β/luciferase reporter plasmid (IFN-β-Luc) and the Renilla luciferase construct phRL-TK and then infected with PEDV (at an MOI of 1 or 0.1, respectively) or mock-infected for 24 h. The cells were retransfected with 1 μg of poly (I:C) as a positive inducer. As shown in Fig. 1a, IFN-β luciferase activity enhanced markedly in positive controls, while it was almost not detected in PEDV-infected IECs. In addition, IFN-β mRNA expression was hardly detected in PEDV-infected IECs similar to mock-infected group, however, it had significant expression in poly (I:C)-transfected group at the indicated times (12 h and 24 h, p < 0.01) (Fig. **1**b). This result was consistent with the luciferase reporter assay. Taken together, PEDV infection of IECs did not induce IFN-β activation.

Increasing evidence showed that viruses not only inhibit the induction of type I IFNs, but also block dsRNA-induced production of type I IFNs to escape the innate immune surveillance of the host [25-27]. To identify whether PEDV was able to inhibit dsRNA-induced IFN-β production, IFN-β-Luc was transfected into PEDV-infected and uninfected cells, respectively. The cells were retransfected with or without poly (I:C) 24 h later. As a result, activation of the IFN-β promoter decreased significantly in poly (I:C)-transfected, PEDV-infected cells compared with mock-infected cells transfected with poly (I:C) (Fig. **1**c). It showed PEDV also inhibited poly (I:C)-mediated IFN-β induction.

### PEDV impeded poly (I:C)-mediated activation of IRF-3, but not NF-κB

**Fig. 2:**
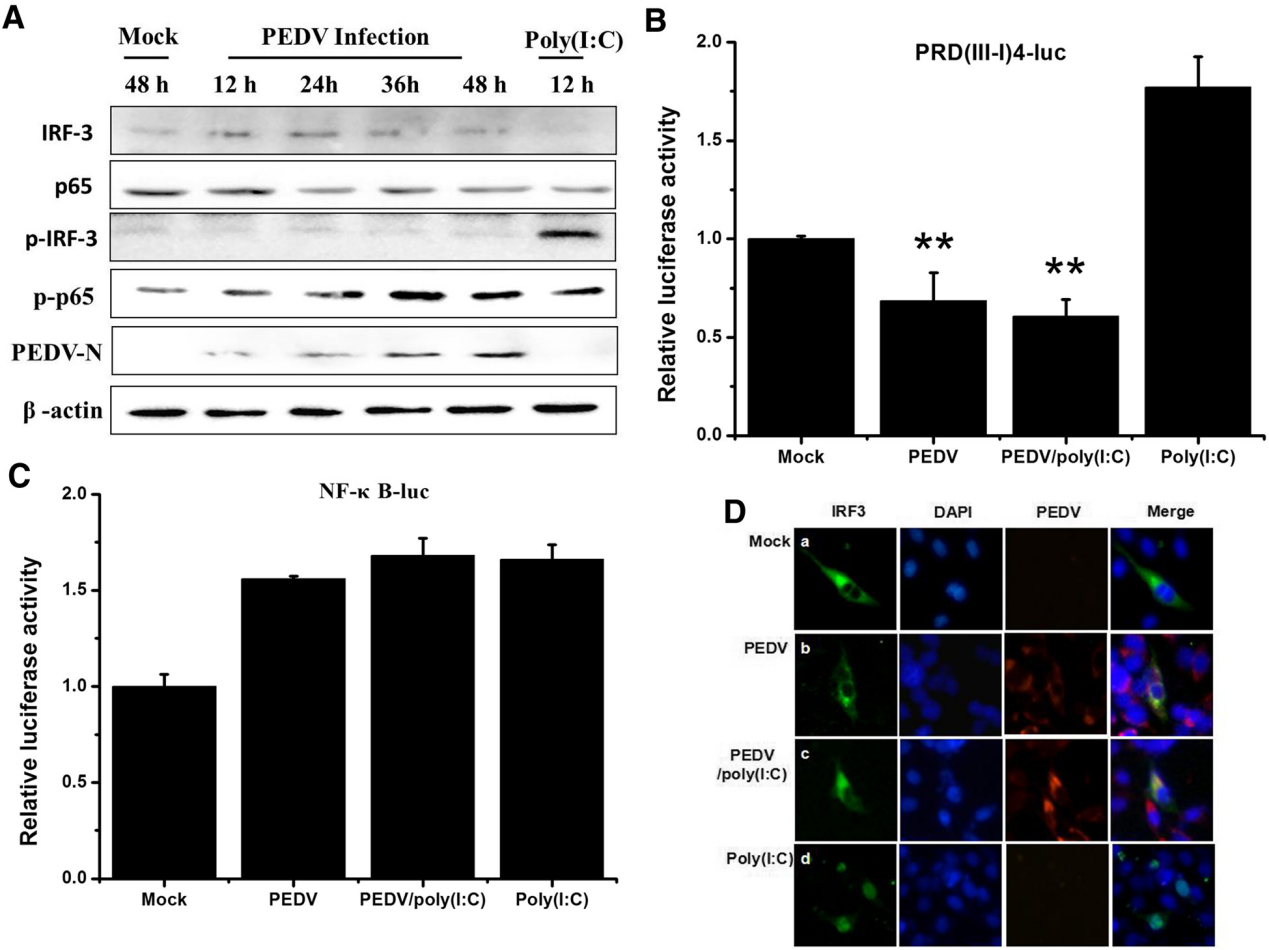
PEDV inhibits dsRNA-mediated IRF-3 activation, but not NF-κB. **a** IECs were infected with PEDV at an MOI of 1 for 12, 24, 36 and 48 h, or treated with poly (I:C) for 12 h. To detect the activation of IRF-3 and p65 after PEDV infection, the cell extracts were prepared at the indicated times and subjected to western blot analysis with antibodies specific for IRF-3, p65, p-IRF-3, p-p65 and PEDV N McAb. Anti-β-actin was included as a control for sample loading. These experiments were performed in duplicate. **b** and **c** IECs were infected or mock-infected with PEDV at an MOI of 1 for 12 h, and then cells were cotransfected with (PRDIII-I) 4-Luc (**b**) or pNF-κB-Luc (**c**) and phRL-TK for additional 24 h. Cells were retransfected with poly (I:C) for 12 h, harvested, and then subjected to a dual-luciferase assay. All data are expressed as means ± SD of 3 independent experiments. **p < 0.01 as compared with poly (I:C). **d** IRF3-GFP fusion protein transfected with IECs and then infected with PEDV at an MOI of 1 and mock-infected cells served as negative controls. 24 h later, cells were retransfected with poly (I:C) (positive control) (**c** and **d**) or untransfected (**a** and **b**) for 12 h. Cells were fixed with 4 % paraformaldehyde, permeabilized with 0.1 % Triton X-100, and stained by DAPI (blue). Cells were incubated with anti-PEDV rabbit polyclonal antibody (red) and TRITC-labeled goat anti-rabbit secondary antibody, then analyzed for fluorescence by confocal microscopy. Magnification, ×20 (Leica, Wetzlar, Germany)

IRF-3 and NF-κB are two essential IFN-β transcription factors. In unstimulated cells, IRF-3 is ubiquitously present in the cytoplasm as an inactive monomer, whereas NF-κB is present as a homodimer or heterodimer bound to the inhibitory proteins IκB in the cytoplasm [28]. Phosphorylation, which is a key step during IRF-3 and NF-κB activation, in turn, leads to nuclear translocation. Therefore, we evaluated whether PEDV infection induced IRF-3 and p65 activation by western blot analysis. Following PEDV infection, the whole cell extracts were prepared for the indicative times, in Fig. 2a, IRF-3 still existed in the cytoplasm, and the levels of IRF-3 protein was almost equal with mock-infected cells, while the phosphorylation of IRF-3 (p-IRF-3) did not detect in the PEDV infected cells in comparision to a obviously signal in poly (I:C)-transfected cells. On the contrary, compared with the amount of NF-κB subunit p65 in the cytoplasm, the phosphorylation of p65 (p-p65) increased with progression of PEDV infection in the nucleus. Meanwhile, the concentration of poly (I:C)-induced p65 nuclear translocation was clearly increased. PEDV N protein was also detected in PEDV-infected cells. These data suggested that PEDV did not induce activation of IRF-3, but NF-κB.

We then used luciferase reporter assay system to determine whether IRF-3 and NF-κB are linked with the inhibition of IFN-β production after PEDV infection. As shown in Fig. 2b, IRF-3 luciferase activity was sharply decreased in PEDV-infected cells, and poly (I:C)-induced IRF-3 activation was also inhibited by PEDV in comparison to a remarkably signal in poly (I:C)-transfected cells. However, in Fig. 2c, compared with mock-infected cells, NF-κB luciferase activity significantly enhanced both in PEDV-infected and poly (I:C)-transfected cells. In addition, we found that poly (I:C)-induced activation of NF-κB was not blocked by PEDV. To further identify PEDV-inhibited poly (I:C)-mediated activation of IRF-3, confocal microscopy assay was used. As a result, IRF3-GFP remained in the cytoplasm of both mock-infected (Fig. 2d. a) and PEDV-infected (Fig. 2d. b) IECs compared with poly (I:C) controls, in which clear translocation to the nucleus was observed (Fig. 2d. d). Furthermore, PEDV could block poly (I:C)-mediated IRF-3 nucleus migration (Fig. 2d. c). Taken together, our date clearly implied that PEDV impeded dsRNA-mediated IFN-β transcription primary by interfering with IRF-3 activation, but not NF-κB.

### PEDV failed to block TBK1/IKKε activity

**Fig. 3:**
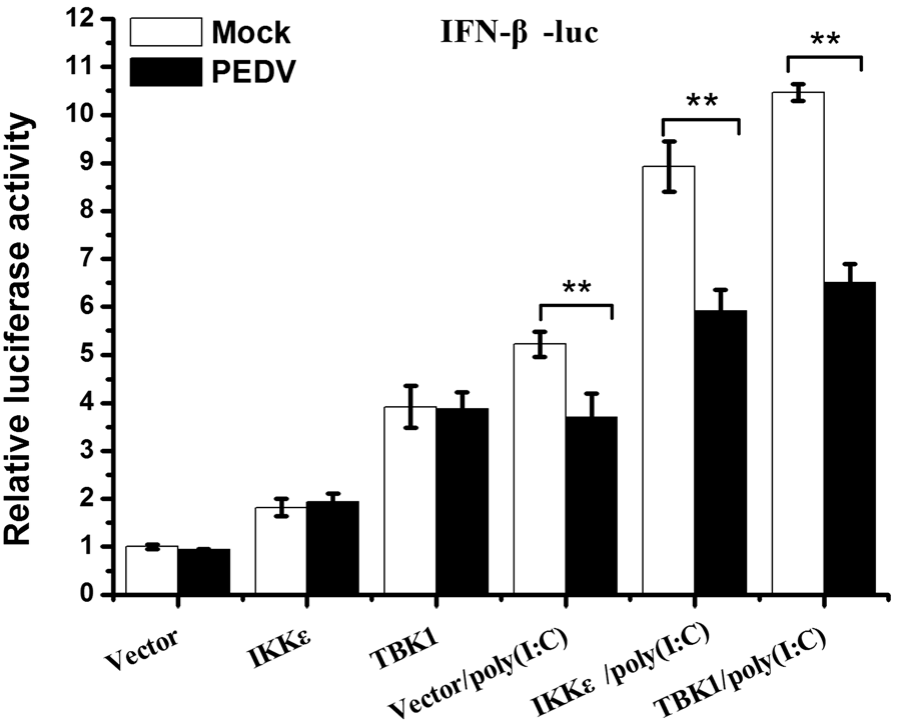
PEDV does not block the activity of TBK1/IKKε. IECs were infected or mock-infected with PEDV at an MOI of 1 for 12 h, and then cells were cotransfected with the IFN-β promoter luciferase reporter and TBK1/IKKε expression plasmids or vector for 24 h. Cells were then retransfected with poly (I:C) as a positive control for 12 h. Cells were harvested and subjected to a dual-luciferase assay. Data were analyzed and the ratio of firefly luciferase expression to Renilla luciferase activity was shown. All data are expressed as means ± SD of 3 independent experiments. **p < 0.01 compared with PEDV-infected, expression plasmids or vector-transfected control

TBK1 and IKKε are essential kinases for the IRF-3 activation [29]. In order to ascertain whether PEDV inhibited poly (I:C)-induced IRF-3 activation by impeding TBK1/IKKε kinase activity, we cotransfected plasmids expressing TBK1/IKKε kinase and a plasmid encoding the IFN-β promoter of the luciferase reporter into infected or mock-infected IECs and retransfected the cells with or without poly (I:C) at 36 h.p.i. As show in Fig. 3, TBK1/IKKε overexpression increased IFN-β promoter activity in both infected and mock-infected IECs, and obviously upregulation was detected in IECs transfected with poly (I:C), suggesting that PEDV failed to block TBK1/IKKε activity. However, poly (I:C)-induced IFN-β promoter activity in IECs overexpression of TBK1/IKKε plasmids was significantly inhibited by PEDV. It showed that PEDV interrupting dsRNA-induced IFN-β production should localize upstream from TBK1/IKKε.

### PEDV inhibited RIG-I-mediated IFN-β production

**Fig. 4:**
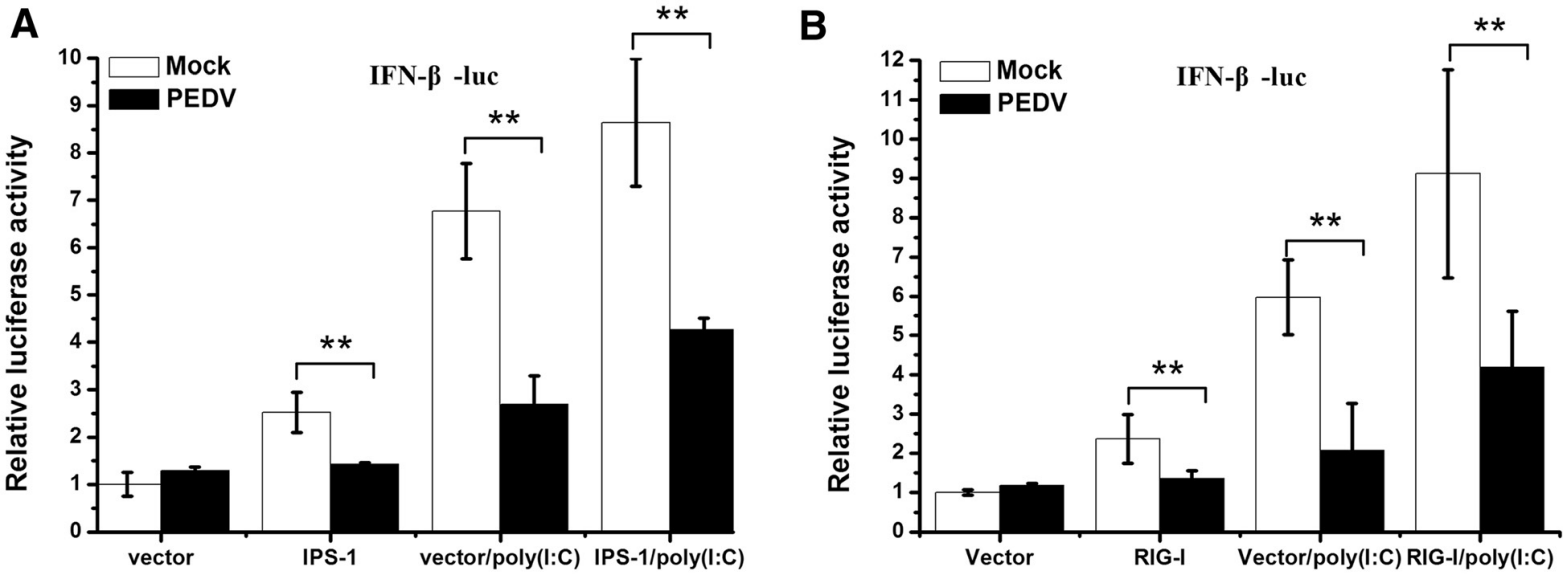
PEDV inhibits IPS-1- and RIG-I-induced IFN-β production. PEDV-infected IECs were cotransfected with the IFN-β promoter luciferase reporter and IPS-1 (**a**) or RIG-I (**b**) expression plasmids or vector for 24 h. Cells were then retransfected with poly (I:C) for 12 h. Cells were harvested and luciferase activity was analyzed using a dual-luciferase assay. All data are expressed as means ± SD of 3 independent experiments. **p < 0.01 compared with PEDV-infected, expression plasmids or vector-transfected control

It is possible that PEDV blocks poly (I:C)-mediated IFN-β production by suppression of the individual molecules upstream of TBK1/IKKε in RIG-I signaling pathway. To explore this possibility, mock- and PEDV-infected IECs were cotransfected with IPS-1 expression plasmid and IFN-β promoter luciferase reporter plasmid, respectively. As shown in Fig. 4a, overexpression of IPS-1 in IECs could enhance IFN-β luciferase activity in mock-infected cells, but it was completely inhibited in PEDV-infected cells. For the poly (I:C) transfection experiments, there appeared significant restriction of IFN-β luciferase expression in PEDV-infected cells compared with that of mock-infected cells. These date indicated that PEDV interacted with IPS-1 to block poly (I:C)-mediated IFN-β transcription.

IPS-1 is an adapter molecule of RIG-I, the data showed that PEDV blocked IPS-1-induced IFN-β production in dsRNA signaling pathway, thus, we speculated that RIG-I-induced IFN-β production in this signaling pathway was also inhibited. To verify it, mock- and PEDV-infected IECs were transfected with RIG-I expression and IFN-β reporter plasmids. The results showed that IFN-β luciferase activity was markedly increased in IECs overexpressing RIG-I, but was completely inhibited by PEDV infection. And the IFN-β reporter signal could be observed in IECs stimulated with poly (I:C), while this signal could be sharply reduced in RIG-I-transfected, poly (I:C)-stimulated and PEDV-infected IECs (Fig. 4b).

## Conclusions

In summary, the findings of the present study suggested that PEDV-infection in IECs inhibits dsRNA-induced IFN-β induction by interfering with IRF-3 activity associated with RIG-I-mediated signaling pathway. The target interaction molecules of PEDV intervention of dsRNA-induced IFN-β production primarily was IPS-1. However, as a limitation to this study, host cells may inhibit or activate multiple signaling pathways simultaneously in response to exogenous stimulus, and some other transcription factors may have been blocked or activated in this process. Here, we only addressed the mechanisms of PEDV-induced inhibition of IFN-β production in relation to the molecules of RIG-I signaling pathways *in vitro*. Further studies are needed. Overall, elucidation of the influence of PEDV evasion of the host innate immune response will aid in the development of antiviral agents to prevent the spread of PEDV during the early infection phase.

## Materials and methods

### Viruses, cells, and reagents

The African green monkey kidney cell line VeroE6 and swine small intestine epithelial cells (IECs) [30, 31] were respectively cultured in Dulbecco’s modified Eagle’s medium (DMEM) and Dulbecco’s modified Eagle’s F12 Ham medium (DMEM-F12) supplemented with 10 % fetal bovine serum at 37 °C in a humidified atmosphere of 5 % CO_2_. PEDV strain CV777 was propagated in VeroE6 cells in DMEM containing 2.5 μg/mL of trypsin.

Poly (I:C) was purchased as a sodium salt (Sigma-Alorch, Saint Louis, MO, USA) and dissolved in water to obtain a stock solution of 10 mg/mL. The Dual-Luciferase® Reporter Assay System was purchased from Promega Corporation (Madison, WI, USA) and monoclonal anti-β-actin antibody was purchased from Sigma-Aldrich (St. Louis, MO, USA). Anti-IRF-3, anti-p65 anti-p-IRF-3 and anti-p-p65 rabbit polyclonal antibodies and secondary horseradish peroxidase (HRP)-conjugated anti-rabbit IgG were purchased from Cell Signaling Technology, Inc. (Beverly, MA, USA). Rhodamine isothiocyanate (TRITC)-labeled goat anti-rabbit IgG were purchased from the Zhongshan Company (Beijing, China). Anti-PEDV rabbit polyclonal antibodies and anti-PEDV N protein monoclonal antibody (McAb) were prepared in our laboratory, which could specifically react with PEDV.

### Plasmids

The plasmids IFN-β-Luc for IFN-β, PRD (III-I) 4-Luc for IRF-3, and pNF-κB-Luc for NF-κB were kindly donated by Dr. Shaobo Xiao (Huazhong Agricultural University, Wuhan, Hubei Province, China) [25]. The pEF-BOS empty vector and pEF-Flag-RIG-I recombinant expression plasmid were kindly provided by T. Fujita (Tokyo Metropolitan Institute of Medical Science, Tokyo, Japan) [32]. The pEF-Bos-Flag-TRIF, pCDNA3-Flag-IKKε and pCDNA3-Flag-TBK1 recombinant expression plasmids, and the pCDNA3 empty vector, and the conjugate IRF3-green fluorescence protein (GFP) expression construct were kindly provided by K. Fitzgerald (University of Massachusetts Medical School, Worcester, MA, USA) [27]. The porcine IPS-1 (NCBI accession No: EU082069.1) gene was cloned from porcine kidney cells by reverse transcription polymerase chain reaction (RT-PCR) using the specific primer pair. The IPS-1 primers were 5′-CCGGGTACCACCATGACGTTTGCCGAGGACAA-3′ and 5′-TTTCTCGAGTCACTGGGGCAGGCGCCGCC-3′. Porcine IPS-1 was inserted into pcDNA3.1 (+) using the restriction enzymes *Kpn*I and *Xho*I.

### Transfection and luciferase assay

IECs were plated in 24-well plates at a density of 1 × 10^5^ cells/well and cotransfected with 0.2 μg of the luciferase reporter plasmids (IFN-β-Luc, [PRDIII-I] 4-Luc, and pNF-κB-Luc, respectively), and the Renilla luciferase construct phRL-TK (Promega Corp.), as an internal control (0.1 μg) with Lipofectamine 2000 reagent (Invitrogen Corp.) according to the manufacturer’s instructions when the cells reached 70 %–80 % confluence. The cells were then infected or mock-infected with PEDV for 24 h. Cells were retransfected with or without poly (I:C) (1.0 μg) for an additional 12 h. Or IECs were infected or mock-infected with PEDV for 12 h prior to transfection the luciferase reporter plasmids alone or cotransfection the indicated expression plasmids (0.5 μg). The cell lysates were harvested and luciferase activity was analyzed using a dual-luciferase assay system and a luminometer (Turner BioSystems, Inc. Sunnyvale, CA, USA) according to the manufacturer’s instructions. Data represent relative firefly luciferase activity normalized to Renilla luciferase activity. The resulting ratios were used to compare the expression of the firefly luciferase gene in PEDV-infected cells to that present in mock-infected cells.

### Real-time PCR amplification of IFN-β

Total RNA was extracted from the transfected cells using TriQuick reagent (Beijing Solarbio Science & Technology Co., Ltd., Beijing, China) according to the manufacturer’s instructions and then reverse-transcribed into complementary DNA (cDNA) using murine leukemia virus reverse transcriptase (GBI Labs/Golden Bridge International, Inc., Mukilteo, WA, USA) with oligo dT random hexamers (HaiGene Technology, Harbin, China). The cDNA was then subjected to real-time PCR with specific primer pairs targeting IFN-β (F: 5′-GCTAACAAGTGCATCCTCCAAA-3′ and R: 5′-CCAGGAGCTTCTGACATGCCA-3′) and β-actin (F: 5′- GGCTCAGAGCAAGAGAGGTATCC-3′, and R: 5′-GGTCTCAAACATGATCTGAGTCATCT-3′. β-actin mRNA was used as an endogenous control.

### Nuclear translocation assay

IECs were seeded in 24-well plates and then transfected with 1 μg of IRF3-GFP fusion expression constructs per well using Lipofectin transfection reagent (Invitrogen Corp.) when cells reached confluence. Cells were then mock-infected or infected with PEDV at a multiplicity of infection (MOI) of 1. At 24 h postinfection, cells were transfected with 1 μg of poly (I:C) or left untransfected. After 12 h, cells were removed from the culture medium and washed three times in cold phosphate-buffered saline (PBS). Next, cells were fixed in 4 % paraformaldehyde for 15 min at room temperature, quenched with 0.1 M glycine for 5 min, and then permeabilized with 0.1 % Triton X-100 for 10 min. Afterward, the cells were incubated with anti-PEDV antibody (dilution, 1:500) for 1 h followed by TRITC-labeled goat anti-rabbit secondary antibody (dilution, 1:200) for 30 min at 37 °C. The nuclei were stained with 4′,6-diamidino-2-phenylindole-dihydrochloride (DAPI) (Invitrogen Corp.). Cells were examined using a TCS SP2 AOBS confocal microscope (Leica Camera AG, Wetzlar, Germany).

### Western blot analysis

IECs were infected with PEDV at an MOI of 1 or treated with poly (I:C) for the indicative times, lysed in 2× sodium dodecyl sulfate (SDS) sample buffer and boiled for 10 min. Whole Cells extracts were separated by 12 % SDS-polyacrylamide gel electrophoresis and transferred to a nitrocellulose membrane, which was blocked with 5 % (w/v) bovine serum albumin (BSA) in tris-buffered saline (10 mM Tris-Cl at pH 7.5 and 150 mM NaCl) containing 0.05 % Tween 20 (TBST) at room temperature for 1 h. The membranes were then incubated with a primary antibody (dilution, 1:1000) at 4 °C overnight and a secondary HRP-conjugated antibody (dilution, 1:2500) for 1 h at room temperature. Protein blots were developed using an enhanced chemiluminescence (ECL) detection system and exposed to X-ray film (Clinx Science Instruments Co., Ltd., Shanghai, China).

### Statistical analysis

All data were expressed as means ± standard deviations (SD) of 3 independent experiments. The statistical significance was tested by student ’s t-test and p-values less than 0.05 were considered statistically significant.
